# Dual immunomodulator therapy with upadacitinib and bimekizumab in severe hidradenitis suppurativa: A case report

**DOI:** 10.1016/j.jdcr.2025.10.066

**Published:** 2025-11-08

**Authors:** Kenan Kherallah, Claire S. Chung, Justin Gillenwater, Edward C. Jones-López, Vivian Y. Shi, Jennifer L. Hsiao, Katrina H. Lee

**Affiliations:** a Keck School of Medicine, University of Southern California, Los Angeles, California; b Division of Plastic and Reconstructive Surgery, Los Angeles General Medical Center/Keck Medicine of USC, Los Angeles, California; c Division of Infectious Disease, Keck School of Medicine, University of Southern California, Los Angeles, California; d Department of Dermatology, University of Washington, Seattle, Washington; e Department of Dermatology, Keck School of Medicine, University of Southern California, Los Angeles, California

**Keywords:** bimekizumab, biologics, hidradenitis suppurativa, small molecule inhibitor, upadacitinib

## Background

Hidradenitis suppurativa (HS) is a skin disease characterized by painful nodules, abscesses, and draining tunnels, commonly affecting intertriginous regions. Its progressive course often leads to scarring, functional impairment, and psychosocial burden.^1^ Management of moderate to severe HS typically involves a multimodal approach including antibiotics, hormonal or metabolic agents, biologics, and procedures often used in sequence or in combination.^2^ Disease severity and treatment response vary widely, and many patients fail multiple lines of treatment, including biologics. This therapeutic challenge reflects the complex immunopathology of HS, which involves dysregulation of inflammatory pathways, including tumor necrosis factor-alpha, interleukin (IL)-17, IL-1, and Janus kinase/signal transducer and activator of transcription (JAK/STAT) signaling.^3^ For recalcitrant cases, there is growing interest in dual immunomodulatory therapy (DIT). We report a case of severe, treatment-refractory HS successfully managed with combined JAK1 and IL-17A/F inhibition.

## Case

A 54-year-old man with Hurley stage III HS, hypertension, and depression presented with HS involving the neck, axillae, abdomen, groin, buttocks, and thighs. Symptoms began at 17 years old, and he was diagnosed with HS at 26 years old. His HS significantly impacted his quality of life, limiting both work and physical activity.

He had previously failed multiple serial single-biologic treatments, including adalimumab 40 mg weekly for 6 months, infliximab 7.5 mg/kg every 4 weeks for 5 months, secukinumab 300 mg every 2 weeks for 3 months, and ustekinumab 90 mg every 8 weeks for 3 months. Systemic antibiotics used alone or in combination included doxycycline, trimethoprim-sulfamethoxazole, cephalexin, clindamycin, rifampin, ciprofloxacin, amoxicillin-clavulanate, cefdinir, metronidazole, and a 16-week course of intravenous ertapenem 1 g daily.

**Fig 1:**
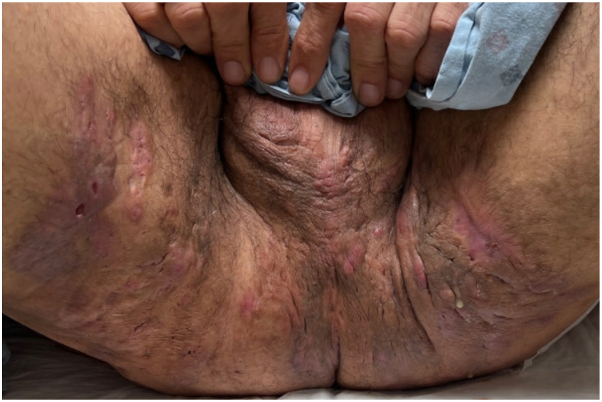
Numerous inflamed nodules and interconnected draining tunnels involving the thighs, inguinal folds, perineum, and scrotum prior to the initiation of upadacitinib.

While on ustekinumab, physical exam revealed abscesses, inflamed nodules, and interconnected draining tunnels involving the thighs, inguinal folds, perineum, and scrotum (Fig 1). Thus, the decision was made to transition the patient to upadacitinib 30 mg once daily, which led to clinical improvement. At the 6-week follow-up on upadacitinib, his Hidradenitis Suppurativa Physician's Global Assessment score improved from 5 to 3, and his pain numerical rating scale decreased from 10 to 5. Despite these improvements, he continued to experience bothersome drainage and pain and was unable to discontinue his oral antibiotics without significant worsening. Therefore, bimekizumab 320 mg subcutaneously every 4 weeks was added 10 months after starting upadacitinib.

**Fig 2:**
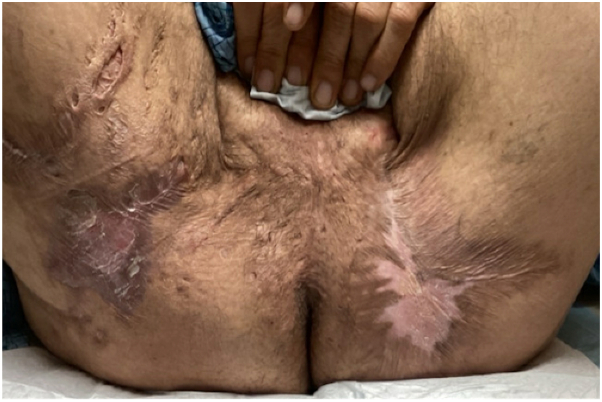
Quiescent nondraining tunnels, atrophic scarring, and postsurgical scarring of the thighs, inguinal folds, perineum, and scrotum after 18 months of dual immunomodulatory therapy.

One month later, the patient reported no new HS lesions and decreased drainage. The patient continued to show improvement in his HS lesions at subsequent visits, with Hidradenitis Suppurativa Physician's Global Assessment and pain numerical rating scale both decreased to 2 on DIT. Eighteen months after starting bimekizumab, the patient described the best disease control he had ever experienced. Physical exam revealed quiescent scars with minimal erythema and no drainage (Fig 2). During DIT, the patient underwent 2 deroofing procedures, one to the right posterior thigh and one to the right buttock/upper thigh. DIT was not interrupted for either procedure, and there were no postoperative complications or surgical site infections.

During combination therapy, the patient developed asymptomatic oral candidiasis 11 months after starting bimekizumab, which resolved with clotrimazole lozenges. Oral thrush recurred 5 months later, resolving with oral fluconazole 150 mg daily for 7 days. No other adverse events occurred. Intermittent laboratory testing was unremarkable, with stable blood counts, platelets, and hemoglobin (nadir of 12.0 g/dL during DIT; 10.7 g/dL prior to DIT). Liver enzymes, renal function, and triglycerides were within normal limits throughout treatment.

## Discussion

Current treatment options for moderate-to-severe HS include 3 Food and Drug Administration-approved biologics: adalimumab, secukinumab, and bimekizumab. Despite these options, patients can remain refractory to treatment. For such individuals, there is growing interest in DIT, but reports of the efficacy of this approach remain limited. Maronese et al recently proposed combining biologic and small-molecule agents as a strategy to overcome monotherapy resistance in HS.^4^ One prior case report described the use of secukinumab and baricitinib in a patient with HS, resulting in 90% ulcer healing and no reported adverse events.^5^ To our knowledge, this is the first reported case of combination therapy with upadacitinib and bimekizumab in a patient with HS.

In this patient, treatment with upadacitinib 30 mg daily resulted in clinical improvement, aligning with positive findings from a phase II trial in which upadacitinib achieved significantly higher rates of Hidradenitis Suppurativa Clinical Response 50 at week 12 compared to historical placebo rates.^6^ However, despite these improvements, the patient continued to experience drainage and still required concomitant oral antibiotics. Bimekizumab was therefore introduced, and the patient achieved an excellent clinical response at 4-week follow-up with continued improvement of his symptoms at 18 months. The patient tolerated DIT well, with hematologic and lipid profiles remaining stable throughout treatment. The patient did develop oral candidiasis during DIT, which resolved with antifungal treatment and did not require treatment discontinuation. It is well known that IL-17 inhibition is associated with increased risk of candidiasis. Larger studies are needed to investigate whether dual therapy confers an added risk of oral thrush.

Emerging molecular data support DIT rationale in refractory HS. Krueger et al demonstrated upregulation of JAK/STAT signaling gene sets in lesional HS skin, with IL-17A and IL-17F–driven inflammation prominent in both lesional and perilesional areas, contributing to sustained inflammation.^7^ Concurrent inhibition of JAK and IL-17 pathways may provide broader immunologic control by modulating distinct yet complementary mechanisms in HS pathogenesis. Supporting this, a case series of 3 patients with refractory psoriatic arthritis and/or psoriasis treated with tofacitinib and either ixekizumab or secukinumab achieved substantial improvement, including minimal disease activity and near-complete skin clearance; the regimen was generally well tolerated, with only 1 case of *Clostridioides difficile* infection requiring temporary interruption of tofacitinib.^8^

In conclusion, we present a case of recalcitrant HS with significant clinical improvement on upadacitinib and bimekizumab for a prolonged treatment period of 18 months with no significant adverse events. This outcome supports further investigation of DIT for treatment-resistant HS, though the immunosuppressive burden warrants caution and close monitoring for infections. Additional studies are needed to clarify the additive benefit that DIT may offer over single immunomodulator therapy and to better define its safety profile.

## Conflicts of interest

Dr Shi is on the board of directors for the Hidradenitis Suppurativa Foundation (HSF), an advisor for the National Eczema Association, is a stock shareholder of Learn Health and has served as an advisory board member, investigator, speaker, and/or received research funding from Sanofi Genzyme, Regeneron, AbbVie, Genentech, Eli Lilly, Novartis, SUN Pharma, LEO Pharma, Pfizer, Incyte, Dermavant, Apogee, MoonLake, Navigator Medicine, Boehringer Ingelheim, Almirall, Alumis, Aristea Therapeutics, Menlo Therapeutics, Dermira, Burt’s Bees, Galderma, Kiniksa, UCB, Ceraclere, Bain Capital, Target-PharmaSolutions, Castle Bioscience, Altus Lab/cQuell, MYOR, Polyfins Technology, GpSkin, and Skin Actives Scientific. Dr Hsiao is on the board of directors for the HS Foundation and has served as an advisor, investigator, and/or speaker for AbbVie, Amgen, Aclaris, Boehringer Ingelheim, Galderma, Incyte, Insmed, Moonlake, Navigator Medicines, Novartis, Pfizer, Sanofi, Regeneron, and UCB. Dr Lee has served as an investigator or advisor for Novartis and Incyte. The other authors have no conflicts of interest to declare.
